# Sustained kidney biochemical derangement in treated experimental diabetes: a clue to metabolic memory

**DOI:** 10.1038/srep40544

**Published:** 2017-01-12

**Authors:** Antonio Anax F. de Oliveira, Tiago F. de Oliveira, Larissa L. Bobadilla, Camila C. M. Garcia, Carolina Maria Berra, Nadja C. de Souza-Pinto, Marisa H. G. Medeiros, Paolo Di Mascio, Roberto Zatz, Ana Paula de M. Loureiro

**Affiliations:** 1 Department of Clinical and Toxicological Analyses, Faculty of Pharmaceutical Sciences, University of São Paulo, Av. Prof. Lineu Prestes 580, Bloco 13 B, CEP 05508-000, São Paulo, Brazil; 2 Center for Research in Biological Sciences & Department of Biological Sciences, Institute of Physical and Biological Sciences, Federal University of Ouro Preto, Campus Morro do Cruzeiro, Ouro Preto, MG, Brazil; 3 Department of Biochemistry, Institute of Chemistry, University of São Paulo, Av. Prof. Lineu Prestes 748, CEP 05508-000, São Paulo, Brazil; 4 Nephrology Division, Department of Internal Medicine, School of Medicine, University of São Paulo, Av. Dr. Arnaldo, 455, 3-s/3342, CEP 01246-903, São Paulo, Brazil

## Abstract

The occurrence of biochemical alterations that last for a long period of time in diabetic individuals even after adequate handling of glycemia is an intriguing phenomenon named metabolic memory. In this study, we show that a kidney pathway is gradually altered during the course of diabetes and remains persistently changed after late glycemic control in streptozotocin-induced diabetic rats. This pathway comprises an early decline of uric acid clearance and pAMPK expression followed by fumarate accumulation, increased TGF-β expression, reduced PGC-1α expression, and downregulation of methylation and hydroxymethylation of mitochondrial DNA. The sustained decrease of uric acid clearance in treated diabetes may support the prolonged kidney biochemical alterations observed after tight glycemic control, and this regulation is likely mediated by the sustained decrease of AMPK activity and the induction of inflammation. This manuscript proposes the first consideration of the possible role of hyperuricemia and the underlying biochemical changes as part of metabolic memory in diabetic nephropathy development after glycemic control.

Diabetes is a major cause of chronic kidney disease, which affects more than 50 million people, thereby exerting a huge impact on health systems, and thus, new prevention and treatment approaches are urgently needed^1^. Although the achievement of normoglycemia reduces the morbidity and mortality risks due to diabetes, a period of exposure to high glucose induces metabolic memory, which results in diabetic individuals continuing to experience vascular complications even after achieving tight glycemic control^2^,^3^. The concept of metabolic memory in diabetes garnered attention following the publication of the Diabetes Control and Complications Trial^4^ and the subsequent Epidemiology of Diabetes Interventions and Complications study^5^,^6^, both of which enrolled type 1 diabetic patients, and of the UK Prospective Diabetes Study^7^, which investigated type 2 diabetic patients and noted the vascular benefits of early intensive glycemic control.

The intriguing occurrence of biochemical alterations that last for a long period of time in diabetic individuals even after adequate handling of glycemia has been the subject of much interest in the last three decades^3^,^4^,^5^,^6^,^7^,^8^,^9^,^10^,^11^,^12^,^13^,^14^,^15^. An understanding of these biochemical changes is crucial to improving therapies aiming to prevent and treat diabetic complications^16^.

Several biochemical pathways have been identified as relevant for the pathogenesis of diabetic kidney disease. Transforming growth factor-β (TGF-β) is a central player in this process because the activation of TGF-β induces the expression of pro-fibrotic proteins and inhibits extracellular matrix degradation by decreasing protease expression^17^,^18^. These effects result in extracellular matrix expansion and lead to glomerulosclerosis and tubulointerstitial fibrosis, both of which are histological hallmarks of advanced diabetic nephropathy^19^.

AMP-activated protein kinase (AMPK) activity plays a key role in inhibiting glomerular extracellular matrix accumulation mediated by TGF-β in diabetes^20^. Kidney tissues isolated from diabetic mice and humans exhibit reduced AMPK activity^20^, which is associated with the upregulation of NADPH oxidase isoform 4 (Nox4) and increased Nox activity, as observed in a diabetic mouse model^21^. Furthermore, a cyclic pathway linking reduced pAMPK expression, Nox4 upregulation, TGF-β1 activation and additional AMPK deactivation has been proposed to induce the extracellular matrix accumulation observed in diabetic kidneys^22^. Recently, You and coworkers^23^ determined that the upregulation of podocyte Nox4 in a transgenic mouse model leads to reduced expression of fumarate hydratase in the kidney, increased fumarate levels in the renal cortex and increased fumarate excretion in the urine. Both *in vitro* and *in vivo*, the increase in fumarate levels or the reduction in fumarate hydratase activity stimulated TGF-β expression, demonstrating a link among Nox4 activity, intermediate metabolism and kidney fibrosis. Opposite effects on fumarate levels and TGF-β expression were observed in the kidneys of a diabetic mouse model treated with a Nox1/Nox4 specific inhibitor^23^.

In this study, we sought to determine whether the above-mentioned components (pAMPK, fumarate and TGF-β) that have been implicated in a pathway closely related to diabetic kidney disease (DKD) development remain altered in the kidneys of diabetic rats after early (four weeks of hyperglycemia) and late (12 weeks of hyperglycemia) glycemic control. We also assessed some other players related to the above-mentioned pathway, such as uric acid clearance, peroxisome proliferator-activated receptor gamma coactivator 1-alpha (PGC-1α) expression, and the mitochondrial DNA epigenetic marks 5-methylcytosine (5-mC) and 5-hydroxymethylcytosine (5-hmC). The data reveal a kidney pathway that is gradually altered during the course of diabetes and that remains persistently changed after late glycemic control and suggest a role for hyperuricemia in hyperglycemic memory.

## Results

**Figure 1:**
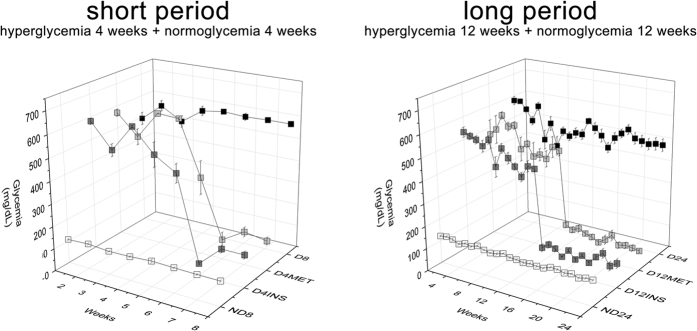
Non-fasting glucose levels (mg/dL) over eight or 24 weeks. Glycemic control was achieved seven to 14 days after treatment initiation. The data are presented as the means ± SEM. N = 5 to 6 animals in the short-period group, N = 6 to 10 animals in the long-period group.

Streptozotocin-induced diabetic rats were maintained at a hyperglycemic status for either four (short period) or 12 weeks (long period). The glycemic levels were then controlled by treatment with insulin alone or insulin combined with metformin (100 mg/kg/day) for four (D4INS and D4MET) or 12 weeks (D12INS and D12MET). The control groups consisted of non-diabetic (ND) and non-treated diabetic (D) animals maintained for eight (ND8 and D8), 12 (ND12 and D12), and 24 weeks (ND24 and D24). The diabetic rats achieved glycemic control seven to 14 days after treatment initiation, showing glycemic levels comparable to those of the non-diabetic control group (Fig. 1). Accordingly, the glycated hemoglobin (HbA1c) levels were increased in the diabetic animals and were reduced after treatment was administered (Table 1). The diabetic animals in the short- and long-period groups presented increased proteinuria after eight, 12, 18 and 24 weeks of hyperglycemia. The treatment of the animals in the short-period group was initiated prior to the onset of proteinuria^24^ and prevented its development. In contrast, the treatment of the animals in the long-period group was initiated after the onset of proteinuria and had completely reversed the observed proteinuria at the end of the 24-week period. Tubular injury was detected after 12 weeks of diabetes by measuring the KIM-1 levels in the urine, and these levels returned to normal following treatment. Hyperglycemia led to augmented kidney/body weight ratios, which also returned to normal once glycemia control was achieved (Table 1).

**Figure 2:**
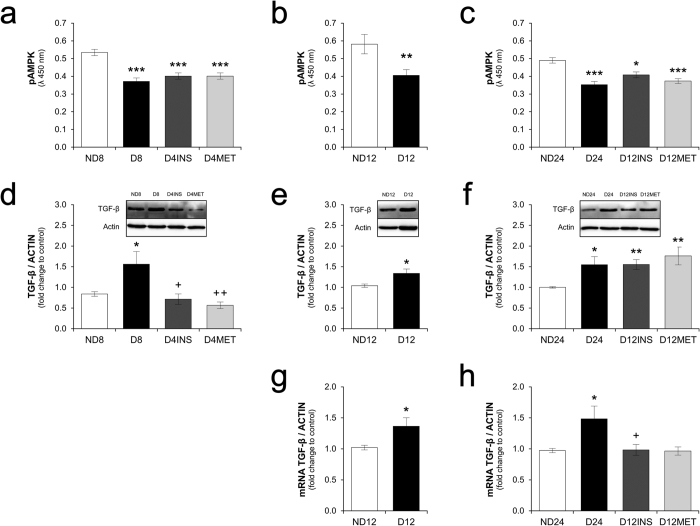
Reduced pAMPK and increased TGF-β expression are early alterations in the diabetic kidney and are not restored after late glycemic control. (**a**,**b** and **c**) pAMPK expression after 8, 12, and 24 weeks was determined by ELISA and is plotted as the intensity of absorbance at 450 nm. (**d**,**e** and **f**) Protein levels of TGF-β after eight, 12, and 24 weeks, expressed as fold changes relative to the control groups, and representative immunoblots obtained for TGF-β analysis at each time point. (**g** and **h**) mRNA levels of TGF-β (ratio of C_T_ values of TGF-β to those of β-actin) after 12 and 24 weeks analyzed by real-time PCR and expressed as fold changes relative to the control groups. The data are expressed as the means ± SEM. *p < 0.05, **p < 0.01, and ***p < 0.001 compared with the respective non-diabetic group. ^++^p < 0.01 compared with the respective diabetic group. N = 5 or 6 animals in the short-period group, N = 6 to 10 animals in the long-period group. The western blot experiments included N = 4 or 5 animals in the short-period group and N = 5 or 6 animals in the long-period group. Full-length blots are presented in Supplemental Figures 1 and 2.

The active phosphorylated AMPK (pAMPK) levels in the kidney were decreased in hyperglycemic animals at all time points evaluated (eight, 12, and 24 weeks) and were not restored four (short period) or 12 weeks (long period) after glycemic and renal function control was achieved (Fig. 2a,b and c). Accordingly, kidney TGF-β protein expression was increased after eight, 12, and 24 weeks of hyperglycemia. Treatments initiated after four weeks of hyperglycemia were able to normalize the TGF-β levels within 8 weeks (short period), but treatments initiated after 12 weeks of hyperglycemia did not reduce the TGF-β protein expression levels until the end of a 24-week period, even though the glycemic levels and renal function were normal (Fig. 2d,e and 2f). We also examined kidney TGF-β1 gene expression and found that it was increased after 12 and 24 weeks of hyperglycemia and that glycemic control reversed this increase (Fig. 2g and h).

**Figure 3:**
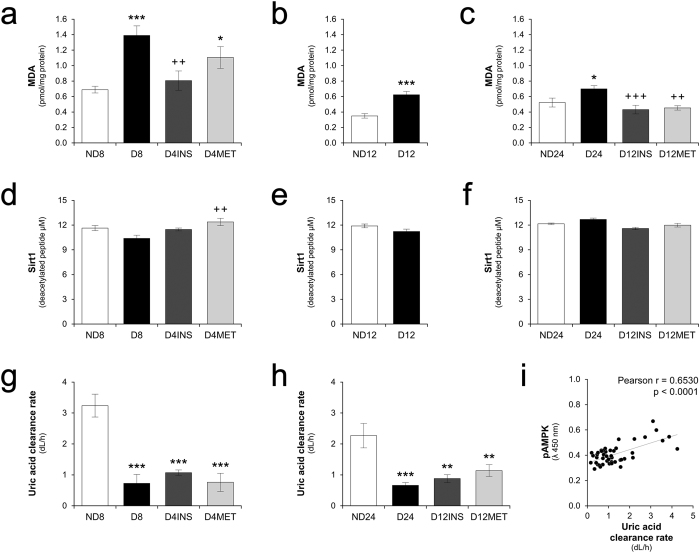
Increased kidney MDA levels were detected in diabetic animals after eight (**a**), 12 (**b**) and 24 weeks (**c**) and were normalized in the animals after the achievement of glycemic control, with the exception of the animals in the short-period group treated with insulin plus metformin. (**d**,**e** and **f**) Sirt-1 activity in kidney samples from the short- and long-period groups. Uric acid clearance was reduced after eight (**g**) and 24 weeks (**h**) of diabetes and was not recovered after glycemic control. A correlation between pAMPK expression and uric acid clearance was observed (**i**). The data are expressed as the means ± SEM. *p < 0.05, **p < 0.01, and ***p < 0.001 compared with the respective non-diabetic group. ^++^p < 0.001 and ^+++^p < 0.001 compared with the respective diabetic group. N = 4 to 6 animals in short-period group, N = 6 to 10 animals in the long-period group. For the Pearson correlation analysis, paired samples of both periods were included (N = 45).

The persistent reduction in the levels of pAMPK could be due to several factors that have already been reported, such as oxidative stress, decreased sirtuin 1 (SIRT1) activity, and/or hyperuricemia^25^,^26^,^27^. We observed that the kidney malonaldehyde levels, which serve as a measure of oxidative stress, were increased in hyperglycemic animals. In the short-period group, treatment with insulin alone reversed this increase, but this reversal was not achieved by treatment with insulin combined with metformin (Fig. 3a). In the long-period group, however, malonaldehyde returned to the normal levels after glycemic control was achieved by either treatment (Fig. 3b and c). Kidney SIRT1 activity in the short-period group was only slightly decreased in the hyperglycemic animals but increased in the diabetic animals that received the metformin treatment (Fig. 3d). No change in SIRT1 activity was observed in kidney samples from the long-period group (Fig. 3e and f). Uric acid clearance, however, accompanied the trend obtained for the kidney pAMPK levels (Fig. 3g and h), and a good positive correlation was noted between these two factors (Fig. 3i). An inverse significant correlation was also observed between plasma uric acid levels and kidney pAMPK expression (r = −0.3856, p = 0.0033).

The energy status was assessed by quantification of kidney AMP, ADP and ATP (Table 2). No difference in AMP/ATP and ADP/ATP ratios was observed between the groups of the short period experiment (8 weeks). However, both treatments (D12INS, D12MET) led to increased kidney AMP/ATP and ADP/ATP ratios in the animals of the long period experiment (24 weeks), not justifying the decreased pAMPK levels.

**Figure 4:**
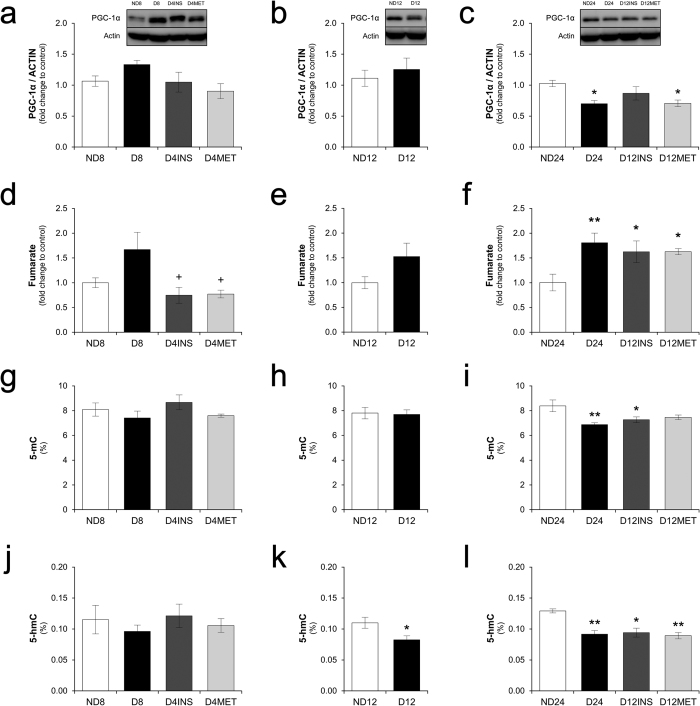
Levels of PGC-1α, fumarate, and mitochondrial DNA methylation and hydroxymethylation are changed during 12 weeks of diabetes and are not normalized after late glycemic control. (**a**,**b** and **c**) Kidney PGC-1α expression after eight, 12, and 24 weeks and representative immunoblots. (**d**,**e** and **f**) Fumarate levels in kidney tissue after eight, 12, and 24 weeks. (**g**,**h** and **i**) Percentage of 5-methylcytosine (5-mC) and (**j**,**k** and **l**) percentage of 5-hydroxymethylcytosine (5-hmC) in mitochondrial DNA after eight, 12, and 24 weeks. The data are expressed as the means ± SEM. *p < 0.05, **p < 0.01, and ***p < 0.001 compared with the respective non-diabetic group. ^+^p < 0.05 and ^++^p < 0.01 compared with the respective diabetic group. N = 5 or 6 animals in the short-period group, N = 5 to 9 animals in the long-period group. The western blot experiments included N = 4 or 5 animals in each group. Full-length blots are presented in Supplemental Figures 3 and 4.

Because the persistent decrease of pAMPK could affect mitochondrial biogenesis and activity^20^, we examined the expression of PGC-1α and some markers of intermediate metabolism. The kidney PGC-1α levels did not change in the short-period group (Fig. 4a), whereas in the long-period group, the reduced PGC-1α expression levels observed after 24 weeks of hyperglycemia were not normalized by the combined insulin/metformin treatment. Animals treated with insulin alone still presented slightly decreased levels of PGC-1α, although significant differences were not observed compared with either the hyperglycemic or non-diabetic control groups (Fig. 4b and c). To examine the kidney intermediate metabolism, pyruvate, lactate, malate, succinate, fumarate, glutamine, and glutamate were quantified (Table 3 and Fig. 4d,e and f). After eight weeks, no significant difference was observed between non-diabetic and hyperglycemic animals (Table 3, Fig. 4d), but a trend towards increased metabolite levels was observed after 24 weeks of hyperglycemia (Table 3). Among the metabolites, only fumarate appeared to have similar levels in the both treated and diabetic groups, which were significantly higher than in the non-diabetic group (Table 3 and Fig. 4f).

PGC-1α activity was linked to the expression of a mitochondrial isoform of DNA methyltransferase 1 (mtDNMT1)^28^. So, we examined kidney mtDNA 5-mC and 5-hmC levels. Although no difference in either the 5-mC or 5-hmC levels was observed in the mtDNA of the animals belonging to the short-period group (Fig. 4g and j), decreased 5-hmC (but not 5-mC) levels were found after 12 weeks of hyperglycemia (Fig. 4h and k), and the levels of both 5-mC and 5-hmC were decreased in the mtDNA of the diabetic and treated diabetic animals belonging to the long-period group (Fig. 4i and l). The sustained decrease in PGC-1α expression, concomitant with fumarate accumulation and the downregulation of mtDNA epigenetic marks, only after late glycemic control, indicate that changes in mitochondrial function during the course of diabetes serve as a contributing factor for hyperglycemic memory.

## Discussion

The early tight management of glycemia in diabetic individuals is of utmost importance for slowing down the progression of DKD^16^. Amazingly, a reversal of DKD has been observed 10 years but not five years after pancreas transplantation in eight long-term type 1 diabetes patients with mild to advanced diabetic nephropathy at the time of transplantation^29^. Notwithstanding the clear possibility of the reversal of diabetic nephropathy after the restoration of euglycemia, the reported studies also demonstrate a long delay between these events^29^, which ultimately shows that the biochemical alterations experienced by the kidney over a long period of loose glycemic control are not promptly reversed by tight glycemic management.

Different molecular components and mechanisms have been proposed to explain the metabolic memory sensed by kidney cells after exposure to hyperglycemia. Persistent oxidative stress^30^, glomerular basement membrane accumulation^31^, expression of extracellular matrix proteins^15^,^32^, and increased p38 MAPK activity^33^ have been confirmed after the normalization of glucose levels in different *in vitro* and *in vivo* models. Additionally, epigenetic mechanisms operating in the pathogenesis of DKD are emphasized as key events underlying metabolic memory, particularly due to their long-term persistence^34^. Indeed, apart from oxidative stress^10^,^35^, epigenetic alterations regulating the expression of proinflammatory genes have been shown to persist in vascular endothelial cells in several experimental models of hyperglycemic memory^11^,^12^,^36^,^37^.

To the best of our knowledge, there is no information regarding a hyperglycemic memory of sequential components of a fibrogenic pathway implicated in DKD development. In this study, we identified a pathway that is gradually altered in the diabetic rat kidney after the restoration of normal glycemia and that depends on the previous period of hyperglycemia experienced by the animal.

First, we found that a four-week period of hyperglycemia is sufficiently long to initiate a metabolic memory of reduced pAMPK expression in the rat kidney, and this reduced pAMPK expression had not returned to the baseline level four weeks after the achievement of glycemic control. The same finding was observed in the animals who underwent 12 weeks of hyperglycemia followed by 12 weeks of glycemic control (Fig. 2a–c). Although it is well known that hyperglycemia triggers a reduced expression of kidney pAMPK^20^, this study provides the first line of evidence demonstrating that the reinstatement of normoglycemia is not sufficient to promptly reverse the kidney pAMPK levels. This effect of hyperglycemia on the sustained downregulation of AMPK activity after glycemic control was previously observed only in bovine retinal capillary endothelial cells (BRECs) *in vitro* and retinas of streptozotocin-induced diabetic rats^26^. Possible drivers of AMPK activation are an increased AMP/ATP ratio and increased activities of the upstream kinases liver kinase B1 (LKB1) and Ca^2+^/calmodulin-dependent protein kinase kinase^26^. The class III NAD^+^-dependent deacetylase sirtuin 1 (SIRT1) has been shown to be a positive upstream regulator of the activity of the LKB1/AMPK pathway in human embryonic kidney 293T cells and other cells^25^. SIRT1 downregulation in response to poly(ADP-ribose)polymerase (PARP) activation in BRECs and rat retinas exposed to high glucose or oxidative stress was crucial to understanding the persistent high levels of molecules related to inflammation (NF-κB), apoptosis (Bax), and damage (PAR) responses observed after restoration of the normal glucose levels, which were effects mediated by the persistently decreased activity of the LKB1/AMPK pathway and increased ROS generation^26^.

Conversely, we observed here that hyperglycemia did not significantly affect kidney SIRT1 activity (Fig. 3d,e and f). This observation does not preclude, however, the possible decrease in SIRT1 activity in some specific cell types, such as proximal tubular cells and glomerular podocytes^38^, an effect that may be diluted in the kidney homogenates used in this study. It is possible to observe a slight, albeit insignificant, decrease in kidney SIRT1 activity after eight weeks of hyperglycemia (Fig. 3d). Nevertheless, the increased kidney SIRT1 activity detected in the diabetic animals that received the metformin treatment (Fig. 3d) and the decreased oxidative stress detected in the treated diabetic animals (Fig. 3a and c) were not accompanied by a recovery of the kidney pAMPK levels. Indeed, contrary to expectations^26^,^39^,^40^,^41^, metformin was unable to restore the kidney pAMPK levels (Fig. 2a and c).

Metformin acts in part by activating AMPK^42^. Different mechanisms, including a mild inhibition of complex 1 of the mitochondrial respiratory chain followed by an increase in the AMP/ATP ratio, activation of LKB1, and inhibition of AMP deaminase, an enzyme in the AMP degradation pathway to uric acid, have been proposed for this activation^43^,^44^. Given its polarity, metformin enters cells through membrane-bound organic cation transporters (OCT)^45^. The human OCT1 and OCT2 isoforms are important for metformin hepatic uptake and secretory renal clearance, respectively^45^. Both Oct1 and Oct2 are expressed in rodent kidneys^45^, with Oct1 and Oct2 constituting ~23% and ~75% of all renal Oct in rats^24^. It has been estimated that 76% of metformin hepatic uptake and 60% of metformin renal uptake are mediated by Oct1 and Oct1/Oct2, respectively, in mice^45^. However, Oct expression on the basolateral surface of the proximal tubules is downregulated in experimental diabetes^24^. In fact, a downregulation of approximately 50% of renal Oct1 and Oct2 protein expression was observed four weeks after the induction of diabetes by streptozotocin in male Sprague-Dawley rats, and this downregulation was not prevented by administration of a low-dose insulin treatment^24^. In a rat model of type 2 diabetes, the renal expression of Oct2 was also decreased to 50% of that found in the control rat kidney^46^. Thus, it is likely that the early reduction in the expression of Oct in the diabetic rat kidney reduces the renal uptake of metformin and its ability to activate kidney AMPK. Higher doses of metformin than that used in this study (100 mg/kg) would likely be necessary to reach the same kidney AMPK activation effect observed in non-diabetic rodent kidney injury models^40^,^41^. We did not find a study that quantified pAMPK in the diabetic rodent kidney when metformin was administered after at least four weeks of hyperglycemia.

Independently of hyperglycemia, hyperuricemia is a factor that also leads to a pronounced downregulation of Oct2 expression in rat kidneys^47^. Decreased renal clearance of uric acid with resultant increases in the plasma or serum uric acid levels have been observed in rats after three or seven weeks of diabetes induced by streptozotocin^48^,^49^. Therefore, we assessed the plasma and urine levels of uric acid (Supplemental Table 1) and found that the decreased renal clearance of uric acid in the hyperglycemic rats was not improved in the treated diabetic animals in both the short- and long-period groups (Fig. 3g and h). In addition to aiding the lack of a metformin effect on kidney pAMPK levels even after 12 weeks of glycemic control, the sustained decrease in uric acid clearance resembled the sustained decrease in kidney pAMPK expression (Fig. 2a–c). Although we cannot establish a link between these events, a good correlation could be demonstrated (Fig. 3i). At least in the liver of mice and in human HepG2 cells, it has been shown that an increased activity of AMP deaminase and a consequent increase in uric acid production, which are observed in diabetes, mediate hepatic gluconeogenesis *via* pAMPK downregulation^27^. The effect of excess plasma uric acid on the kidney pAMPK levels still needs to be investigated.

An elevation of serum uric acid in type 1 diabetes has been found to be an independent predictor for overt diabetic nephropathy development^50^ and it has been indicated as an important and novel player in this process^51^,^52^. Mild hyperuricemia in rats, corresponding to a 1.5- to 2-fold increase in serum uric acid induced by the uricase inhibitor oxonic acid, causes renal interstitial fibrosis after seven weeks, with increased interstitial collagen deposition and macrophage infiltration but no deposition of uric acid crystals. Fibrosis was prevented by allopurinol administration concomitant with the oxonic acid-containing diet^53^. The role of asymptomatic hyperuricemia in inducing renal fibrosis in patients with DKD has been evidenced in these patients by an increase in urinary TGF-β1 after allopurinol withdrawal^54^. The persistent decrease in uric acid clearance observed in this study in diabetic rats with adequate glycemic control provides the first line of evidence showing that this effect might be an important factor in the metabolic memory of diabetes leading to DKD.

A crucial role for AMPK activity in regulating mitochondrial function and matrix accumulation in diabetic kidneys has been demonstrated in different mouse models of diabetes^20^. Reduced AMPK activity in the mouse diabetic kidney (16 weeks of hyperglycemia) has been linked to reduced mitochondrial biogenesis, reduced pyruvate input into the Krebs cycle, and reduced mitochondrial superoxide radical generation concomitant with increased expression of glomerular fibronectin, type IV collagen, and TGF-β^20^. The decreased pAMPK expression was also localized to the glomeruli, as shown by immunofluorescence^20^. All of these deleterious effects observed in diabetic animals were found to be reversed by the activation of AMPK through the i.p. administration of 5-aminoimidazole-4-carboxamide-1-β-D-ribofuranoside (AICAR)^20^. Here we did not perform immunostaining for pAMPK localization in the rat kidney. Other researchers have demonstrated, by immunohistochemistry, increased TGF-β expression in both tubular and glomerular compartments in rodent models after 8, 12 and 24 weeks of diabetes^55^,^56^,^57^. Since TGF-β is downstream to the other components of the fibrogenic pathway we studied, it is likely that the changes we observed are also present in glomerular and tubular compartments. Indeed, we consider that once we were able to detect the changes in the whole tissue lysate, they may not be limited to a single kidney compartment.

In this study, we observed that the sustained reduction in kidney pAMPK expression was accompanied by a sustained increase in TGF-β protein expression in the long-period group, despite the normalization of TGF-β1 gene expression, the achievement of glycemic control and the optimization of renal function. In contrast, the normalization of glycemia in the short-period group reversed the increase in TGF-β protein expression induced by hyperglycemia (Fig. 2d,e and f). Thus, other elements in addition to the reduction in AMPK activity appear to be necessary for the sustained high expression of the TGF-β protein, as considered below.

The upregulation of TGF-β under high-glucose conditions is well described^58^,^59^,^60^,^61^. Regarding its role in hyperglycemic memory, male Lewis rats maintained in a hyperglycemic state for two weeks or four months (~16 weeks) and thereafter subjected to strict glycemic control by pancreatic islet transplantation (for an additional 12 months or four months, respectively) exhibited normal glomerular TGF-β1 gene expression, although increased TGF-β1 gene expression was observed after four, eight or 12 months of hyperglycemia. However, a point of no return was reached by islet transplantation after eight months (~32 weeks) of hyperglycemia, and glomerular TGF-β1 gene expression remained increased until the end of the study (12 months following the onset of diabetes), accompanied by increases in the gene expression of fibronectin and collagen IV, urinary albumin excretion, proteinuria, and mesangial expansion^62^. The researchers did not assess the level of TGF-β protein expression.

*In vitro* experiments with human proximal tubular epithelial cells have demonstrated that a short period of exposure to high-glucose conditions stimulates TGF-β1 transcription, but the mRNA was poorly translated^63^. Subsequent stimulation of the cells with macrophage-derived cytokine platelet-derived growth factor^63^ or interleukin-1β^64^ stabilized the TGF-β1 mRNA and synergistically increased its translational efficiency, leading to sustained *de novo* synthesis of TGF-β1 protein^63^,^64^. One possible explanation for this effect is that glucose primes the kidney for TGF-β1 protein synthesis through an outside stimulus^63^. Renal macrophage infiltration is an early event in streptozotocin-induced diabetic rats^65^ and has been demonstrated to occur in patients with type 2 diabetes^66^. Hyperuricemia appears to contribute to kidney inflammation mediated by the NOD-like receptor protein 3 (NLRP3) inflammasome, resulting in increased interleukin-1β and interleukin-18 expression in STZ-induced diabetic rats^49^. Urate-lowering agents, such as allopurinol and quercetin, suppress renal NLRP3 inflammasome activation, thereby increasing the protection of diabetic rats against kidney injury^49^. Therefore, we hypothesize that the persistent hyperuricemia observed in this study may play a role in the persistent elevation of the TGF-β protein levels in the kidneys of the treated diabetic animals of the long-period group.

Because the reduced kidney AMPK activity is associated with reduced mitochondrial biogenesis and function^20^, we assessed whether PGC-1α expression, epigenetic marks (5-mC and 5-hmC) in mitochondrial DNA, and some intermediate metabolites (pyruvate, lactate, malate, succinate, fumarate, glutamine, and glutamate) are persistently altered in the kidneys of the treated diabetic rats. As observed for the kidney TGF-β protein expression, persistent changes occurred only after late glycemic control (Fig. 4 and Table 3).

PGC-1α is a co-activator of the forkhead box O (FoxO) family of transcription factors, acting as a master regulator of oxidative metabolism and mitochondrial biogenesis^67^. PGC-1α activity has also been linked to the expression of a mitochondrial isoform of DNA methyltransferase 1 (mtDNMT1)^28^. In this study, we observed a persistent decrease in kidney PGC-1α expression concomitant with a sustained downregulation of mtDNA 5-mC after late glycemic control (Fig. 4c and i).

The methylation of mtDNA is poorly understood, and levels of mtDNA 5-mC in the range of 2 to 5% have been reported in rodent and human fibroblasts^68^,^69^. Shock and coworkers^28^ showed that mtDNMT1 translocates to mitochondria, binds to mtDNA proportionally to the density of CpG dinucleotides, and regulates mitochondrial gene expression^28^. The hypermethylation of mtDNA has been observed in retinal endothelial cells exposed to high-glucose conditions and in the retinal microvasculature from human donors with diabetic retinopathy^70^. The hypomethylation of mtDNA in the liver of high fructose-fed rats has been proposed as a novel underlying mechanism of metabolic syndrome^71^. In this study, we assessed the global content of kidney mtDNA 5-mC, and to the best of our knowledge, this manuscript provides the first report of mtDNA methylation in the diabetic kidney.

Mitochondrial DNA hydroxymethylation (5-hmC) was first described in 2011 by Shock and coworkers^28^, and its function in the mitochondrial genome is not yet clear. A proposed pathway for mtDNA hydroxymethylation is the direct addition of 5-hydroxymethyl groups to cytosine residues driven by mtDNMT1^28^,^72^. In this case, our observation of a persistent downregulation of mtDNA 5-hmC (Fig. 4k and l) could also be a consequence of a reduction in the mtDNMT1 levels due to a decrease in PGC-1α expression. Yamazaki and co-workers^71^ reported reduced levels of mitochondrial 5-hmC and 5-mC in the liver of high fructose-fed rats compared with control rats^71^. Further research on the implications of decreases in mtDNA 5-mC and 5-hmC for DKD development is warranted.

**Figure 5:**
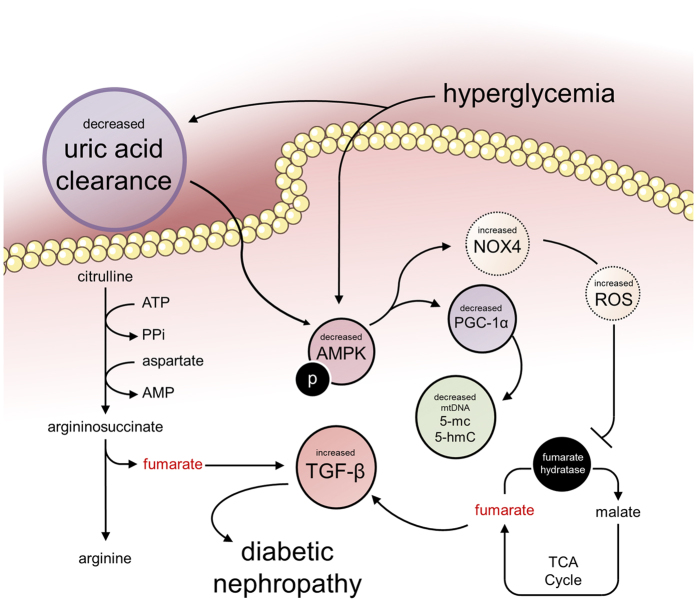
Hyperglycemia triggers a kidney fibrogenic pathway that remains altered after the restoration of normal glycemia depending on the previous period of hyperglycemia experienced by the animal. The colored components of the pathway were quantified in this study and remained altered after late glycemic control, indicating that these constitute part of the diabetic kidney metabolic memory. The persistent disruption of uric acid clearance may feed the prolonged kidney biochemical alterations observed after tight glycemic control. The demonstration of this sustained metabolic derangement may shed light on the understanding of the long interval required between the acquisition of glycemic control in diabetic individuals and reducing the risk of developing DKD.

Among the intermediate metabolites analyzed, fumarate was noted as the only one to maintain its same high level in diabetic rats without and with adequate late glycemic control for 12 weeks. During the period of insulin insufficiency, an increase in amino acid catabolism, as well as increased activity of the urea cycle in the liver and rapid arginine consumption, is expected. The kidney plays a key role in the synthesis of arginine from citrulline to maintain the body arginine pool for non-urea cycle functions. The synthesis pathway consumes ATP and aspartate and releases inorganic pyrophosphate, AMP and fumarate^73^; therefore, this pathway is a possible source of the high kidney fumarate levels observed in the hyperglycemic animals. Early glycemic control, but not late glycemic control, apparently restored the normal metabolism. According to recent data^21^,^22^,^23^, the pathway comprising high glucose → reduced AMPK activity → increased Nox4 → reduced fumarate hydratase activity → increased fumarate levels → increased TGF-β expression plays an important role in the development of diabetic kidney disease. We found that components of this pathway (reduced AMPK activity, increased fumarate levels, and increased TGF-β expression) constitute part of the diabetes metabolic memory (Fig. 5).

Overall, the present study showed that a short period of hyperglycemia (four weeks) induced persistent decreases in uric acid clearance and kidney pAMPK levels during the subsequent four-week period of glycemic control. The rats subjected to a longer period of hyperglycemia (12 weeks) experienced further kidney metabolic alterations that were not reversed in the subsequent 12 weeks of glycemic control, such as fumarate accumulation, increased TGF-β protein expression, decreased PGC-1α expression, and downregulation of mtDNA methylation and hydroxymethylation, and these effects were concomitant with sustained decreases in uric acid clearance and pAMPK levels. The observed changes were grouped in the pathway shown in Fig. 5. Because hyperuricemia is an independent risk factor for diabetic nephropathy development^50^,^51^,^52^,^53^,^54^,^74^, the observed sustained decrease in uric acid clearance in treated diabetes may feed the prolonged kidney biochemical alterations observed after tight glycemic control is achieved. Further interventional research to assess the role of excess plasma uric acid in sustaining the biochemical alterations found in the diabetic kidney in the present study using, for example, allopurinol is needed. Another question raised by the findings obtained in this study is the possible interaction between hyperglycemia and poor uric acid clearance in the induction of metabolic alterations that lead to diabetic complications, which would increase the risk of complications in diabetic subjects with loose glycemic control. The monitoring of the plasma uric acid levels in large clinical trials aimed at evaluating the risk of complications under different targets of glycemic control may aid the understanding of some gaps in the phenomenon of diabetic metabolic memory.

## Methods

### Animals

Eight-week-old male Wistar rats were maintained under a 12-h light/12-h dark cycle and had access to standard laboratory diet and water *ad libitum*. For diabetes induction, streptozotocin (40 mg/kg) freshly dissolved in saline was injected into the penile vein. The control animals received saline intravenously. The diabetic rats were maintained in their hyperglycemic state for either four (short period) or 12 weeks (long period). The animals were then treated with insulin alone or insulin combined with 100 mg/kg metformin (Alcon Biosciences, Mumbai, India) administered by gavage for the subsequent four weeks (D4INS and D4MET, respectively) or in drinking water for the subsequent 12 weeks (D12INS and D12MET, respectively) and were then euthanized. The daily dose of metformin was selected based on the results reported by Zheng and coworkers^26^. Age-matched non-diabetic (ND) and non-treated diabetic (D) controls were euthanized after eight (ND8 and D8), 12 (ND12 and D12) and 24 (ND24 and D24) weeks. The insulin treatment of the animals in the short-period group consisted of 5 to 6 UI of NPH insulin (Lilly, Indianapolis, IN, USA) administered subcutaneously in two daily doses. The insulin treatment of the animals in the long-period group consisted of 2 UI of NPH insulin (Lilly, Indianapolis, IN, USA) in the morning and 2 or 3 UI of glargine insulin (Sanofi, Gentilly, France) at the end of the day. The non-treated diabetic animals of the long-period group received 1 to 3 UI of NPH insulin on alternate days to maintain their glycemic levels under 500 mg/dL and avoid suffering and death before 24 weeks. The body weight and glycemia, measured in the tail vein blood with a Breeze^TM^ 2 glucometer (Bayer, Leverkusen, Germany), were monitored weekly for the duration of the study. Twenty-four-hour urine collections were performed at different time points to assess renal function. At the end of the experiments, the animals were euthanized, and the kidneys were collected and immediately frozen at −80 °C for further analyses. All of the procedures were approved by the Animal Research Ethics Committee of the Faculty of Pharmaceutical Sciences of the University of São Paulo, São Paulo, Brazil (Protocol CEUA FCF/363), and conducted according to the guidelines of the National Council for Animal Experimentation Control (*Conselho Nacional de Controle de Experimentação Animal* – CONCEA, Ministry of Science, Technology, Innovation and Communications, Brazil).

### Protein and Albumin Levels in Urine

The total urine protein levels were quantified using an automated analyzer with a commercial assay (Labmax^®^, Labtest, Lagoa Santa, Brazil). The albumin levels were determined by ELISA (Nephrat, Exocell, Philadelphia, PA, USA) following the manufacturer’s instructions.

### Glycated Hemoglobin

Hemolysates were prepared as described by Jeppson and co-workers^75^. Then, an aliquot of 50 μg of total hemoglobin was subjected to enzymatic cleavage with 0.5 μg of endoproteinase Glu-C (Sigma Aldrich, St. Louis, MO, USA) in a final volume of 100 μL of the digestion buffer (50 mM ammonium acetate, pH 4.3) at 37 °C for 18 h. The digestion was stopped by freezing the samples at −20 °C. After digestion, the samples were centrifuged at 5,600 *g* for 2 min, and aliquots of 15 μL were injected into an UHPLC system (Shimadzu, Kyoto, Japan) interfaced with an Ion Trap mass spectrometer (model AmaZon Speed, Bruker Daltonics, Billerica, MA, USA). All analyses were performed in the positive-ion mode (electrospray interface, ESI^+^), and the ESI-MS parameters (nebulizer, 15 psi; dry gas, 8.0 L/min; dry temperature, 250 °C; capillary 4500 V; enhanced resolution 8100 *m*/*z* s^−1^) were optimized by directly infusing the heptapeptide standard solutions (Merck Millipore, Billerica, MA, USA) into the mass spectrometer. The standard solutions were synthetic heptapetides corresponding to the *n*-terminal sequence of the rat hemoglobin β-chain (V-H-L-T-D-A-E). MS-extracted ion chromatograms were generated using the ions *m/z* 392.2 and *m/z* 473.2 corresponding to the double-protonated heptapeptides (non-glycated and glycated, respectively). The chromatographic conditions used were a 150 × 2.0 mm i.d., 3.0 μm, 100 A Luna C18(2) column (Phenomenex, Torrance, CA, USA) eluted with a gradient of 0.025% TFA in water (solution A) and acetonitrile containing 0.023% TFA (solution B) at a flow rate of 200 μL/min and a temperature of 35 °C as follows: 0 to 3 min, 7% B; 3 to 19 min, 7–20% B; 19 to 21 min, 20–100% B; 21 to 25 min, 100% B; 25 to 27 min, 100–7% B; and 27 to 37 min, 7% B. The calibration curves for the glycated and non-glycated heptapeptides were in the ranges of 0.0015 to 0.03 μg and 0.03 to 0.3 μg, respectively, in the injection volume. The method was in-house validated prior to its use for the quantification of experimental samples.

### Urine Kidney Injury Molecule 1 (KIM-1)

The KIM-1 levels in urine samples were quantified by ELISA (Abcam, Cambridge, UK) according to the manufacturer’s instructions.

### Malonaldehyde Quantitation

Kidney homogenates were prepared by mixing 50 mg of kidney tissue with 500 μL of phosphate buffer saline and 72 μL of butylhydroxytoluene 0.2% in a mechanically driven homogenizer. A 100 μL aliquot of kidney homogenate was used for MDA quantitation. Protein-bound MDA was released by the addition of 10 μL of 4 M sodium hydroxide to the homogenate, and the mixture was incubated at 60 °C for 1 h. The proteins were then precipitated by the addition of 150 μL of 1% sulfuric acid, and the samples were centrifuged at 9,300 *g* for 10 min. Finally, 25 μL of dinitrophenylhydrazine (1 mg/mL in 2 M hydrochloric acid) were added to 175 μL of the supernatant, and the mixture was incubated at room temperature and protected from light for 30 min. Aliquots of 100 μL were injected into a HPLC-DAD system (Shimadzu, Kyoto, Japan). The chromatographic conditions used consisted of a 250 × 4.6 mm i.d., 5.0 μm Luna C18(2) column (Phenomenex, Torrance, CA, USA) that was eluted with a gradient of water (solution A) and acetonitrile (solution B), both containing 0.2% acetic acid, at a flow rate of 1 mL/min and a temperature of 30 °C as follows: 0–28 min, 20–100% B; 28–30 min, 100–20% B; and 30–40 min, 20% B. The DAD detector was fixed to 306 nm to detect the MDA-DNPH product. The calibration curves were in the MDA range of 0.25 to 12 μM.

### TGF-β and PGC-1α Protein Expression

The proteins in kidney tissue were extracted using RIPA buffer containing 10 μL/mL protease and phosphatase inhibitors (Thermo Scientific, Waltham, MA, USA). Fifty micrograms of proteins were electrophoresed on an SDS-PAGE gel and then transferred to a PVDF membrane using a semi-dry method (Trans-Blot^®^ Turbo^TM^, Bio-Rad, Hercules, CA, USA). The membrane was blocked with 5% milk for 1 h at room temperature and then incubated overnight at 4 °C with TGF-β (1:500 Abcam, Cambridge, UK) or PGC-1α (1:1000 Sigma Aldrich, St. Louis, MO, USA) primary antibody. HRP-conjugated secondary antibody (1:2000, Abcam, Cambridge, UK) was incubated for 2 h at room temperature. Chemiluminescent detection was performed by adding the HRP substrate (Millipore, Billerica, MA, USA). Anti-β-actin peroxidase-conjugated antibody (1:10.000 Sigma Aldrich, St. Louis, MO, USA) was used as an endogenous control.

### TGF-β Gene Expression

The total RNA from kidney tissues was isolated using the RNeasy^®^ Mini Kit (Qiagen, Hilden, Germany), and its purity and integrity were confirmed using a Bioanalyzer 2100 system (Agilent Technologies, Santa Clara, CA, USA). Two μg of RNA was converted to cDNA using a High-capacity cDNA Reverse Transcription kit (Applied Biosystems, NJ, USA). For real-time PCR, the cDNA was diluted to 50 ng/μL, and forward and reverse primers, Taqman^®^ Gene Expression Master Mix (Life Technologies, Carlsbad, CA, USA), and RNAse-free water were then added to the mixture. The following primers obtained from Life Technologies were used: Rn00572010_m1 for *Tgf-β1* and Rn00667869_m1 for actin as an endogenous control. The reaction was performed in an ABI Prism^®^ 7500 Sequence Detection System (Applied Biosystems, NJ, USA) under the following conditions: 50 °C for 2 min, 95 °C for 10 min, and 40 cycles of 15 s at 95 °C and 60 °C for 1 min. The gene expression levels were determined by comparing the C_T_ values for *Tgf-β1* relative to those of the actin gene.

### pAMPK Expression

Phospho-AMPKα (Thr172) expression in kidney samples was analyzed using a sandwich ELISA kit (Cell Signaling, Danvers, MA, USA). Kidney homogenates were prepared by mixing 50 mg of renal tissue and 500 μL of lysis buffer with 1 mM phenylmethanesulfonyl fluoride. Aliquots containing 250 μg proteins were diluted in sample buffer, and the assay was conducted following the manufacturer’s recommended protocol.

### Sirtuin 1 Activity

Sirtuin 1 activity in kidney homogenates was measured using a fluorometric assay kit (Fluor-de-Lys, Enzo Life Sciences, Farmingdale, NY, USA) following the manufacturer’s recommended instructions.

### Intermediate Metabolism Assessment

Sample preparation was performed as described by Rahman and coworkers^76^, with some modifications. The analytical system consisted of an Agilent 1200 series HPLC (Wilmington, DE, USA) interfaced with a Linear Quadrupole Ion-Trap mass spectrometer (Model 4000 QTRAP, Applied Biosystems/MDS Sciex Instruments, Foster City, CA, USA). The analyses were conducted with electrospray ionization in the negative mode (ESI^−^, [M-H]^−^), employing the following optimized parameters: CUR, 18 psi; GS1, 60 psi; GS2, 50 psi; CAD, low; TEM, 600 °C; EP, −10 V; IS, −4500 V. The fragmentations and energies used in the MRM mode are described in Supplemental Table 2. The calibration curves contained malate and fumarate (0.5–100 pmol), pyruvate (10–1000 pmol), lactate (25–4000 pmol), succinate (1–130 pmol), glutamine and glutamate (25–400 pmol), as well as 30 pmol of the internal standard ([^13^C_10_^15^N_5_]ATP) in the injection volume. The chromatographic conditions were as follows: A C18(2) Luna column (15 × 2.0 mm i.d., 3.0 μm, 100A, Phenomenex) was eluted with an aqueous solution of 0.1% tributylamine and 0.03% acetic acid (solution A) and 10% of solution A in acetonitrile (solution B) at a temperature of 35 °C and a flow rate of 175 μL/min (0–22 min, 10–100% B; 22–25 min, 100% B; 25–26 min, 100–10% B; and 26–37 min, 10% B). A valve was configured to allow entrance of the eluents into the mass spectrometer in the range of 1.5 to 18 min.

### Uric Acid Quantitation

Aliquots of 50 μL of plasma or urine were diluted in 1,200 μL of water and centrifuged at 20,000 *g* for 5 min. Ten microliters of plasma or 2 μL of urine were injected into a HPLC-DAD system (Shimadzu, Kyoto, Japan). The chromatographic conditions consisted of a 250 × 4.6 mm i.d., 5.0 μm C18 (2) column (Shimadzu, Kyoto, Japan) that was eluted with a gradient of 5 mM ammonium formate (solution A) and methanol (solution B) at a flow rate of 680 μL/min and a temperature of 20 °C as follows: 0–6 min, 0% B; 6–7 min, 0–2% B; 7–9 min, 2–10% B; 9–15 min, 10–80% B; 15–20 min, 80% B; 20–21 min, 80–0% B; and 21–38 min, 0% B. Chromatograms were acquired at 290 nm for uric acid quantitation.

### AMP, ADP, and ATP Quantitation

Samples were prepared as described for intermediate metabolism assessment. Then, 6 μL were injected into the same HPLC-ESI-MS system used for glycated hemoglobin quantitation. The chromatographic conditions used were a 150 × 2.0 mm i.d., 3.0 μm, 100 A Luna C18(2) column (Phenomenex, Torrance, CA, USA) eluted with a gradient of 0.1% tributylamine and 0.03% acetic acid in water (solution A) and acetonitrile (solution B) at a flow rate of 200 μL/min and a temperature of 40 °C as follows: 0 to 10 min, 20–90% B; 10 to 11 min, 90–20% B; 11 to 21 min, 20% B. A valve was configured to allow entrance of the analytes into the mass spectrometer in the range of 2.8 to 12 min. The analyses were conducted with electrospray ionization in the negative mode (ESI^−^, [M-H]^−^), employing the following optimized parameters: nebulizer, 15 psi; dry gas, 8.0 L/min; dry temperature, 200 °C; capillary 3500 V. MS-extracted ion chromatograms were generated using the following transitions: *m/z* 346.0 → *m/z* 210.5 for detection of AMP; *m/z* 426.0 → *m/z* 327.6 for detection of ADP; *m/z* 506.0 → *m/z* 407.6 for detection of ATP; and *m/z* 521.0 → *m/z* 422.6 for detection of [^13^C_10_^15^N_5_]ATP. The calibration curves contained AMP and ADP (1–80 pmol), and ATP (50–1600 pmol), as well as 60 pmol of the internal standard ([^13^C_10_^15^N_5_]ATP) in the injection volume.

### Mitochondrial DNA 5-Methylcytosine (5-mC) and 5-Hydroxymethylcytosine (5-hmC) Assessment

Mitochondrial DNA (mtDNA) from kidney tissue was isolated as described by Stepanov and Hecht^77^. The absence of contamination with nuclear DNA was confirmed by real-time polymerase chain reaction (PCR). The primers used to amplify the ND4 gene were: ND4-F: 5′-TAGCTCAATCTGCCTACGCC-3′ and ND4-R: 5′-ATGGCTGTGATGACTAGGGC-3′. Amplified DNA was labeled with SYBR Green I provided in the SYBR Green PCR Master Mix (Life Technologies, Carlsbad, CA, USA), to so facilitate quantitative detection of PCR products in a reaction volume of 25 μL. The PCR procedure was an initial 10 min at 95 °C, followed by 40 cycles of denaturation for 15 s at 95 °C, annealing for 30 s at 60 °C, and polymerization for 30 s at 72 °C.

Aliquots containing 40 μg of mtDNA were enzymatically hydrolyzed by the addition of 2.85 μL of 200 mM Tris-HCl/MgCl_2_ buffer (pH 7.4) and 1.6 μL (0.5 unit) of deoxyribonuclease I from bovine pancreas (Sigma Aldrich, Saint Louis, MO, USA). The samples were incubated at 37 °C for 1 h, and 1.6 μL (0.00025 unit) of phosphodiesterase I from *Crotalus atrox* (Sigma Aldrich, St. Louis, MO, USA) and 2 μL (1.6 units) of alkaline phosphatase from bovine intestinal mucosa (Sigma Aldrich, St. Louis, USA) were then added. After incubation for 1 h at 37 °C, the samples were centrifuged at 9,300 *g* for 10 min, and aliquots of 60 μL of the supernatant were injected into a HPLC-DAD system for quantification of 2′-deoxycytidine (dC). The chromatographic conditions were a 250 × 4.6 mm i.d., 5.0 μm Luna C18(2) column (Phenomenex, Torrance, CA, USA) that was eluted with a gradient of water (solution A) and 50% methanol (solution B), both containing 0.1% formic acid, at a flow rate of 1 mL/min and a temperature of 35 °C as follows: 0–26.5 min, 0–40% B; 26.5–32 min, 40–75% B; 32–33 min, 75% B; and 33–34 min, 75–0% B. Chromatograms were acquired at 260 nm. Hydrolyzed DNA aliquots containing 0.4 nmol of dC were then taken for 5-mC and 5-hmC quantification. After the addition of 2.25 μL of [^15^N_5_]1,*N*^6^-etheno-2′-deoxyadenosine ([^15^N_5_]1,*N*^6^-εdAdo; 1 pmol/μL) as an internal standard, synthesized according to Loureiro and coworkers^78^, the samples were vacuum dried for 30 min at 40 °C in a miVac concentrator (Genevac, Suffolk, UK) and then suspended in 15 μL of acetonitrile, and 4 μL from the final volume were injected into the same HPLC-ESI-MS/MS system used for intermediate metabolism assessment. The analyses were conducted with electrospray ionization in the positive mode (ESI^+^, [M+H]^+^), employing the following optimized parameters: CUR, 15 psi; GS1, 45 psi; GS2, 50 psi; CAD, low; TEM, 600 °C; EP, 10 V; IS, 5500 V. The fragmentations and energies used in the MRM mode are described in Supplemental Table 3. The calibration curves for dC, 5-mC and 5-hmC were in the range of 5 to 400 pmol, 0.5 to 16 pmol, and 0.01 to 2 pmol, respectively. The chromatographic conditions were the following: A *Syncronis* HILIC column (100 mm × 4.6 mm, 5 μm, Thermo Scientific, Wilmington, DE, USA) was eluted with a gradient of acetonitrile (solution A) and ammonium acetate buffer (pH 8.2, solution B) at a flow rate of 600 μL/min and a temperature of 35 °C (0–20 min, 2–24% B; 20–21 min, 24–2% B; and 21–35 min, 2% B). A valve was configured to allow entrance of the eluents into the mass spectrometer in the range of 9–20 min.

### Statistics

The data are presented as the means ± SEM. The variables that assumed a normal distribution were compared among groups by a two-tailed t test or one-way ANOVA with Sidak’s or Dunnett’s multiple comparisons test. The other variables were analyzed by a Mann-Whitney test or Kruskal-Wallis with Dunn’s multiple comparisons test. Differences with *P* values < 0.05 were considered significant.

## Additional Information

**How to cite this article:** Oliveira, A. A. F. *et al*. Sustained kidney biochemical derangement in treated experimental diabetes: a clue to metabolic memory. *Sci. Rep.* **7**, 40544; doi: 10.1038/srep40544 (2017).

**Publisher's note:** Springer Nature remains neutral with regard to jurisdictional claims in published maps and institutional affiliations.

## Supplementary Material

Supplementary Information

## Figures and Tables

**Table 1:** Early renal function impairment is recovered by glycemic control after both short and long periods of hyperglycemia.

	Groups	Weeks	Body weight (g)	Blood glucose (mg/dL)	HbA1c (%)	Urine Protein (mg/24 h)	Urine Albumin (mg/24 h)	KIM-1 (pg/24 h)	Kidney/body weight ratio
Short-period	**ND8**	*4*	394.1 ± 10.4	107.9 ± 0.6	2.2 ± 0.1	8.2 ± 0.4	—	—	—
		*8*	415.8 ± 10.9	113.2 ± 4.2	2.2 ± 0.1	16.6 ± 3.9	—	—	0.36 ± 0.01
	**D8**	*4*	358.9 ± 10.2*	539.1 ± 21.0*	7.2 ± 0.2*	14.3 ± 2.8	—	—	—
		*8*	348.4 ± 10.5*	590.0 ± 9.6*	8.0 ± 0.2*	34.8 ± 2.4*	—	—	0.63 ± 0.02*
	**D4INS**	*4*	351.4 ± 10.5*	511.7 ± 26.2*	7.0 ± 0.2*	16.1 ± 3.2	—	—	—
		*8*	408.2 ± 13.4+	207.8 ± 18.3+	3.6 ± 0.1*+	16.5 ± 3.8^+^	—	—	0.42 ± 0.02+
	**D4MET**	*4*	336.7 ± 6.8*	560.5 ± 13.1*	7.2 ± 0.2*	9.5 ± 2.2	—	—	—
		*8*	369.2 ± 6.4*	210.8 ± 34.3+	4.7 ± 0.3*+	9.3 ± 3.6+	—	—	0.48 ± 0.03+
Long-period	**ND12**	*12*	449.7 ± 10.7	118.5 ± 4.0	1.8 ± 0.1	8.9 ± 1.4	6.2 ± 1.0	3157 ± 666.2	0.34 ± 0.01
	**D12**	*12*	383.5 ± 8.1*	453.8 ± 21.4*	4.3 ± 0.2*	35.9 ± 8.4*	39.9 ± 8.6*	29722 ± 6343*	0.55 ± 0.02*
	**ND24**	*6*	462.2 ± 10.7	113.8 ± 2.6	1.8 ± 0.1	11.0 ± 1.2	7.8 ± 1.4	—	—
		*18*	530.9 ± 15.0	118.9 ± 2.2	1.9 ± 0.1	11.8 ± 0.7	2.6 ± 0.7	—	—
		*24*	558.8 ± 14.3	114.3 ± 2.1	1.9 ± 0.1	8.8 ± 2.7	5.5 ± 1.6	2878 ± 731.9	0.32 ± 0.01
	**D24**	*6*	358.3 ± 7.6*	457.4 ± 23.5*	5.0 ± 0.2*	16.0 ± 3.2	20.6 ± 5.1	—	—
		*18*	396.5 ± 7.4*	515.5 ± 19.2*	4.5 ± 0.2*	44.8 ± 7.3*	31.7 ± ± 10.1*	—	—
		*24*	410.9 ± 8.6*	497.7 ± 26.9*	4.6 ± 0.3*	36.6 ± 7.2*	21.7 ± 4.4*	17266 ± 1829*	0.60 ± 0.03*
	**D12INS**	*6*	357.8 ± 6.9*	448.0 ± 29.0*	5.3 ± 0.2*	8.4 ± 1.7	14.1 ± 1.7	—	—
		*18*	466.4 ± 13.1*+	102.3 ± 6.9+	2.3 ± 0.1+	25.4 ± 2.0+	18.7 ± 4.4	—	—
		*24*	467.5 ± 13.9*+	107.9 ± 20.1+	2.1 ± 0.1+	5.2 ± 1.6+	2.9 ± 0.5+	4341 ± 1856+	0.40 ± 0.01+
	**D12MET**	*6*	388.0 ± 10.4*	430.0 ± 53.9*	4.6 ± 0.1*	9.7 ± 1.9	11.9 ± 3.7	—	—
		*18*	484.6 ± 23.1+	101.0 ± 19.6+	2.2 ± 0.1+	21.9 ± 5.5+	23.3 ± 8.7	—	—
		*24*	499.5 ± 22.0*+	93.2 ± 7.1+	2.2 ± 0.1+	4.8 ± 0.8+	2.5 ± 0.6+	1238 ± 694.7+	0.40 ± 0.02+

The body weight and blood glucose were assessed weekly, but the values in the table refer to the measurement at the specified time points. For the short-period group, renal function parameters and HbA1c were assessed at two time points (four and eight weeks). For the long-period group, these parameters were assessed at three time points (six, 18 and 24 weeks). The data are presented as the means ± SEM. ^*^p < 0.05 compared with the respective non-diabetic group ^+^p < 0.01 compared with the respective diabetic group. N = 5 to 6 animals in the short-period group, N = 6 to 10 animals in the long-period group.

**Table 2:** Levels of AMP, ADP, ATP, and the ratios AMP/ATP and ADP/ATP in kidney tissue.

	Groups	ATP	ADP	AMP	ADP/ATP	AMP/ATP
8 weeks	**ND8**	0.22 ± 0.01	0.10 ± 0.01	0.03 ± 0.004	0.46 ± 0.03	0.14 ± 0.01
	**D8**	0.18 ± 0.03	0.09 ± 0.01	0.02 ± 0.002	0.48 ± 0.02	0.18 ± 0.06
	**D4INS**	0.19 ± 0.01	0.09 ± 0.008	0.03 ± 0.005	0.48 ± 0.03	0.18 ± 0.03
	**D4MET**	0.22 ± 0.01	0.10 ± 0.007	0.03 ± 0.004	0.46 ± 0.03	0.13 ± 0.02
		*p* = *0.3435*	*p* = *0.7834*	*p* = *0.5051*	*p* = *0.8953*	*p* = *0.5301*
24 weeks	**ND24**	0.34 ± 0.05	0.15 ± 0.04	0.10 ± 0.03	0.38 ± 0.03	0.22 ± 0.02
	**D24**	0.38 ± 0.07	0.16 ± 0.02	0.09 ± 0.01	0.48 ± 0.09	0.25 ± 0.05
	**D12INS**	0.45 ± 0.10	0.36 ± 0.10	0.60**++ ± 0.19	0.80***++ ± 0.06	1.21***+++ ± 0.16
	**D12MET**	0.40 ± 0.09	0.37 ± 0.06	0.55*+ ± 0.13	1.00***+++ ± 0.07	1.39***+++ ± 0.10
		*p* = *0.7890*	*p* = *0.0114*	*p* = *0.0006*	*p* < *0.0001*	*p* < *0.0001*

The data are presented as the means ± SEM in units of pmol/μg of protein. ^*^p < 0.05, ^**^p < 0.01 and ^***^p < 0.001 compared with the respective non-diabetic group. ^+^p < 0.05, ^++^p < 0.01 and ^+++^p < 0.001 compared with the respective diabetic group. N = 5 to 6 animals in the short-period group, N = 6 to 10 animals in the long-period group.

**Table 3:** The fumarate levels are not normalized after late glycemic control.

	Groups	Pyruvate	Lactate	Malate	Succinate	Fumarate	Glutamine	Glutamate
8 weeks	**ND8**	10.33 ± 1.26	32.34 ± 3.37	7.21 ± 0.55	0.35 ± 0.06	1.51 ± 0.15	2.17 ± 0.48	2.20 ± 0.31
	**D8**	7.86 ± 0.42	33.61 ± 3.76	6.35 ± 0.78	0.72 ± 0.15	2.52 ± 0.53	2.97 ± 0.37	2.29 ± 0.27
	**D4INS**	4.11***+ ± 0.49	21.10*+ ± 1.02	4.19**+ ± 0.32	0.39 ± 0.06	1.13+ ± 0.25	2.04 ± 0.26	1.75 ± 0.15
	**D4MET**	6.16** ± 0.55	23.47 ± 1.40	4.65** ± 0.25	0.37 ± 0.11	1.16+ ± 0.12	1.94 ± 0.14	1.59 ± 0.09
		*p* = *0.0003*	*p* = *0.0081*	*p* = *0.0013*	*p* = *0.0646*	*p* = *0.0103*	*p* = *0.1435*	*p* = *0.1097*
24 weeks	**ND24**	15.78 ± 2.36	60.88 ± 8.28	12.97 ± 2.25	3.48 ± 0.45	7.32 ± 1.06	2.94 ± 0.56	1.85 ± 0.35
	**D24**	21.34 ± 1.45	104.14** ± 9.79	18.59 ± 2.22	4.03 ± 0.86	13.19** ± 1.11	3.38 ± 0.29	2.11 ± 0.18
	**D12INS**	19.40 ± 1.99	47.82+++ ± 7.09	10.36+ ± 1.78	1.46+ ± 0.27	11.86* ± 1.46	2.22 ± 0.33	1.39 ± 0.20
	**D12MET**	25.12* ± 2.13	66.71+ ± 8.51	15.46 ± 1.72	2.71 ± 0.44	11.88* ± 0.41	3.82 ± 0.37	2.37 ± 0.22
		*p* = *0.0328*	*p* = *0.0004*	*p* = *0.0491*	*p* = *0.0298*	*p* = *0.0029*	*p* = *0.0662*	*p* = *0.0709*

The data are presented as the means ± SEM in units of pmol/μg of protein. ^*^p < 0.05 and ^**^p < 0.01 compared with the respective non-diabetic group. ^+^p < 0.05 and ^+++^p < 0.001 compared with the respective diabetic group. N = 5 to 6 animals in the short-period group, N = 6 to 10 animals in the long-period group.
