# Supplementary material for: Sustained kidney biochemical derangement in treated experimental diabetes: a clue to metabolic memory
Source: Sci Rep. 2017 Jan 12;7:40544. doi: 10.1038/srep40544 (PMC5228190; doi:10.1038/srep40544)

**Sustained kidney biochemical derangement in treated experimental diabetes: a clue to metabolic memory**

Antonio Anax F. de Oliveira^1^, Tiago F. de Oliveira^1^, Larissa L. Bobadilla^1^, Camila C. M. Garcia^2^, Carolina Maria Berra^3^, Nadja C. de Souza-Pinto^3^, Marisa H. G. de Medeiros^3^, Paolo Di Mascio^3^, Roberto Zatz^4^, and Ana Paula de M. Loureiro^1*^

^1^Department of Clinical and Toxicological Analyses, Faculty of Pharmaceutical Sciences, University of São Paulo, Av. Prof. Lineu Prestes 580, Bloco 13 B, CEP 05508-000, São Paulo, Brazil

^2^Center for Research in Biological Sciences & Department of Biological Sciences, Institute of Physical and Biological Sciences, Federal University of Ouro Preto, Campus Morro do Cruzeiro, Ouro Preto, MG, Brazil

^3^Department of Biochemistry, Institute of Chemistry, University of São Paulo, Av. Prof. Lineu Prestes 748, CEP 05508-000, São Paulo, Brazil

^4^Nephrology Division, Department of Internal Medicine, School of Medicine, University of São Paulo, Av. Dr. Arnaldo, 455, 3-s/3342, CEP 01246-903, São Paulo, Brazil

*Corresponding author: Phone: ++ (55) 11 30911192. E-mail: [apmlou@usp.br](mailto:apmlou@usp.br)

**Supplemental Table 1. Uric acid levels in plasma and urine samples from animals in the short (eight weeks)- and long (24 weeks)-period groups. The data are expressed as the means ± SEM. The *p* values were obtained by ANOVA. *p<0.05 compared with the respective non-diabetic group, as determined according to Dunnett’s multiple comparisons test. N = 5 or 6 animals in the short-period group, N = 6 to 8 animals in the long-period group.**

|  | **Groups** | **Plasma Uric Acid** (mg/dL) | | |  | **Urine Uric Acid** (mg/24h) | | |
| --- | --- | --- | --- | --- | --- | --- | --- | --- |
|  |  |  |  |  |  |  |  |  |
|  |  |  | | |  |  | | |
| Short-period | **ND8** | 0.779 | ± | 0.083 |  | 2.430 | ± | 0.179 |
|  | **D8** | 1.899* | ± | 0.282 |  | 1.153* | ± | 0.230 |
|  | **D4INS** | 1.232 | ± | 0.165 |  | 1.348* | ± | 0.250 |
|  | **D4MET** | 1.544* | ± | 0.136 |  | 1.120* | ± | 0.404 |
|  |  | *p =* 0.0024 | | |  | *p =* 0.0148 | | |
|  |  |  |  |  |  |  |  |  |
| Long-period | **ND24** | 1.567 | ± | 0.226 |  | 3.126 | ± | 0.127 |
|  | **D24** | 3.109* | ± | 0.411 |  | 1.821* | ± | 0.061 |
|  | **D12INS** | 3.202* | ± | 0.392 |  | 2.625 | ± | 0.261 |
|  | **D12MET** | 2.509 | ± | 0.523 |  | 2.437* | ± | 0.177 |
|  |  | *p =* 0.0399 | | |  | *p* < 0.0001 | | |
|  |  |  | | |  |  | | |

**Supplemental Table 2. Fragmentation and energies used for the quantification of intermediate metabolites in kidney tissue by HPLC-ESI-MS/MS. DP = dissociation potential; CE = collision energy; CXP = collision cell exit potential.**

| **Target** | **Q1** (amu) | **Q3** (amu) |  | **Dwell Time** (msec) | **DP** (V) | **CE** (V) | **CXP** (V) |
| --- | --- | --- | --- | --- | --- | --- | --- |
|  |  |  |  |  |  |  |  |
| Pyruvate | 87.0 | 43.2 |  | 50 | -28 | -12 | -6 |
|  |  |  |  |  |  |  |  |
| Lactate | 89.1 | 43.0 |  | 50 | -28 | -20 | -6 |
|  |  |  |  |  |  |  |  |
| Malate | 132.9 | 115.1 |  | 50 | -35 | -16 | -3 |
|  | 132.9 | 70.9 |  | 50 | -35 | -23 | -3 |
|  |  |  |  |  |  |  |  |
| Succinate | 116.9 | 72.9 |  | 50 | -30 | -16 | -11 |
|  | 116.9 | 98.9 |  | 50 | -30 | -16 | -11 |
|  |  |  |  |  |  |  |  |
| Fumarate | 115.0 | 71.0 |  | 50 | -35 | -15 | -11 |
|  | 115.0 | 59.0 |  | 50 | -35 | -15 | -9 |
|  |  |  |  |  |  |  |  |
| Glutamine | 145.1 | 126.9 |  | 50 | -45 | -15 | -10 |
|  | 145.1 | 101.0 |  | 50 | -45 | -19 | -9 |
|  |  |  |  |  |  |  |  |
| Glutamate | 145.9 | 128.1 |  | 50 | -45 | -17 | -5 |
|  | 145.9 | 102.0 |  | 50 | -45 | -19 | -9 |
|  |  |  |  |  |  |  |  |
| [^13^C_10_^15^N_5_]-ATP | 521.2 | 78.9 |  | 50 | -75 | -48 | -5 |
|  |  |  |  |  |  |  |  |

**Supplemental Table 3.** Fragmentation and energies used for the quantification of 5-methylcytosine and 5-hydroxymethylcytosine in kidney tissue by HPLC-ESI-MS/MS. DP = dissociation potential; CE = collision energy; CXP = collision cell exit potential.

| **Target** | **Q1** (amu) | **Q3** (amu) |  | **Dwell Time** (msec) | **DP** (V) | **CE** (V) | **CXP** (V) |
| --- | --- | --- | --- | --- | --- | --- | --- |
|  |  |  |  |  |  |  |  |
| dC | 228.0 | 112.0 |  | 200 | 36 | 15 | 6 |
|  |  |  |  |  |  |  |  |
| 5-mC | 242.0 | 126.0 |  | 200 | 36 | 15 | 6 |
|  |  |  |  |  |  |  |  |
| 5-hmC | 258.0 | 142.0 |  | 200 | 36 | 15 | 6 |
|  |  |  |  |  |  |  |  |
| [^15^N_5_]1,*N*^6^-εdAdo | 281.0 | 165.0 |  | 200 | 41 | 27 | 8 |
|  |  |  |  |  |  |  |  |

**Supplemental Figure 1.** Full-length blots of TGF-β and actin expression for the short period experiment. The red boxes highlight the cropped bands shown in the Figure 2D. Images with the detected bands and the molecular weight ladder are shown separately. TGF-β was detected at 35 kDa and actin was detected at 42 kDa.


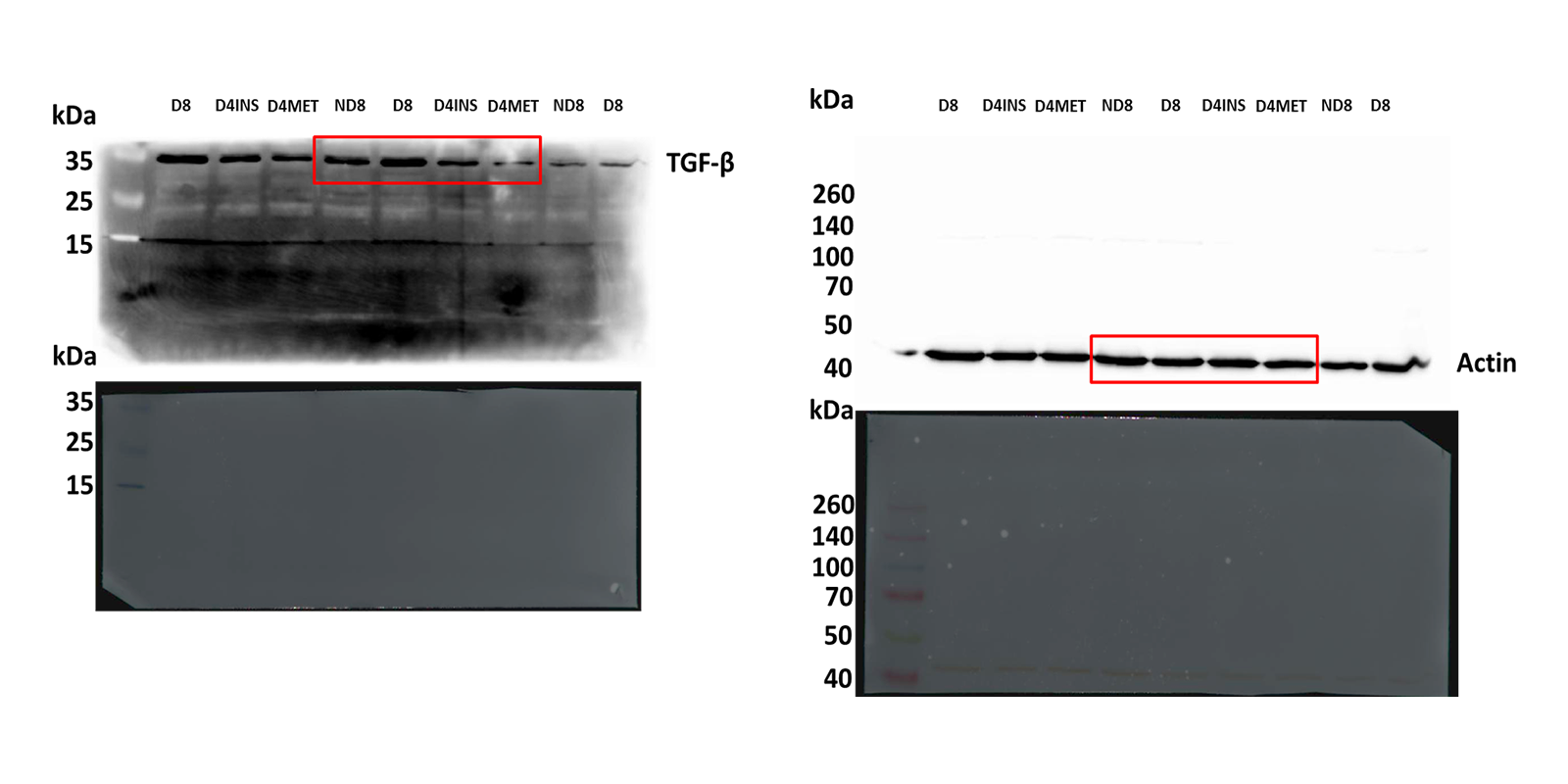


**Supplemental Figure 2.** Full-length blots of TGF-β and actin expression for the long period experiment. The red boxes highlight the cropped bands shown in Figures 2E and 2F. Images with the detected bands and the molecular weight ladder are shown separately. TGF-β was detected at 35 kDa and actin was detected at 42 kDa.


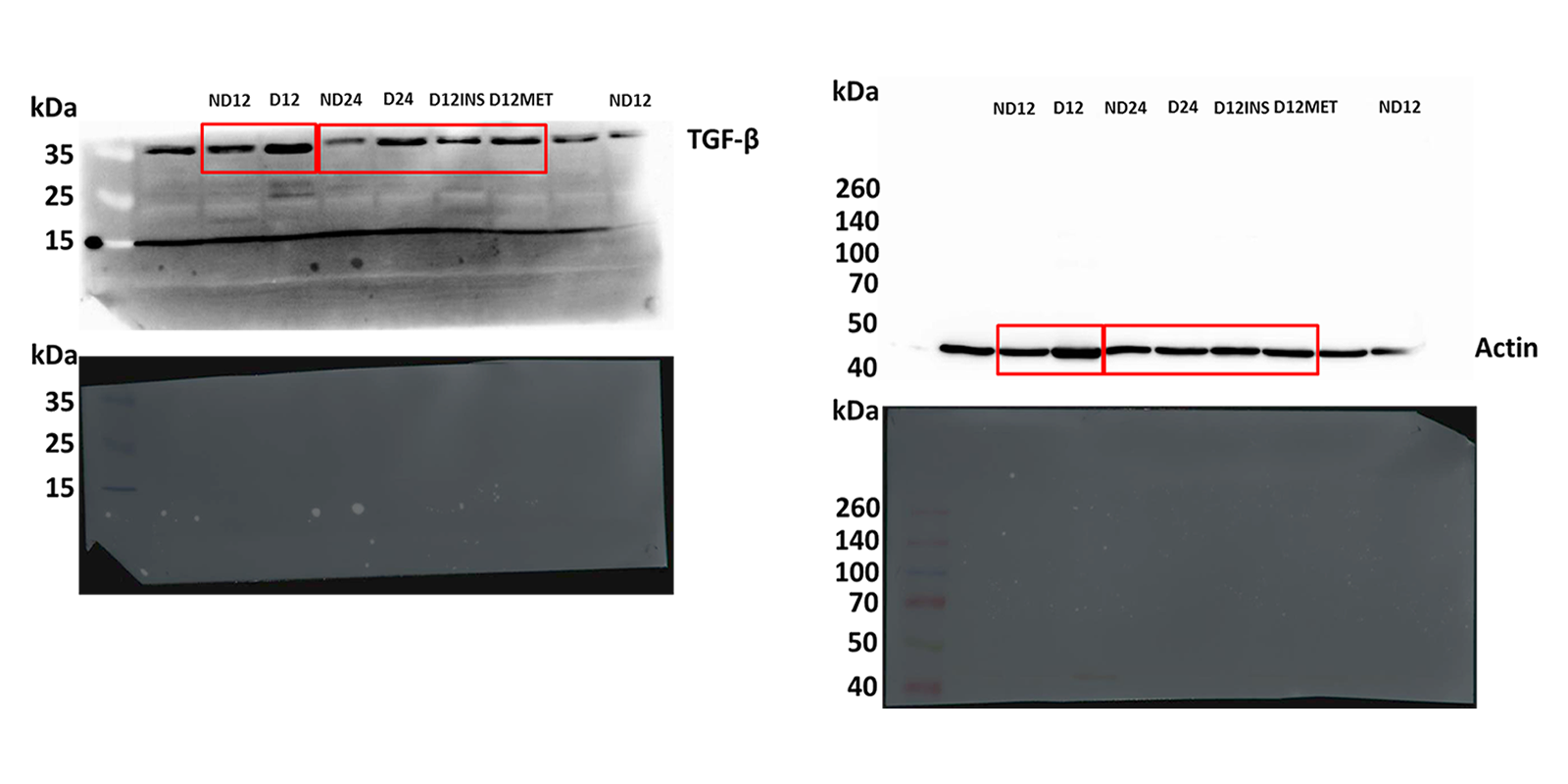


**Supplemental Figure 3.** Full-length blots of PGC-1α and actin expression for the short period experiment. The red boxes highlight the cropped bands shown in the Figure 4A. PGC-1α was detected at 90 kDa and actin was detected at 42 kDa.


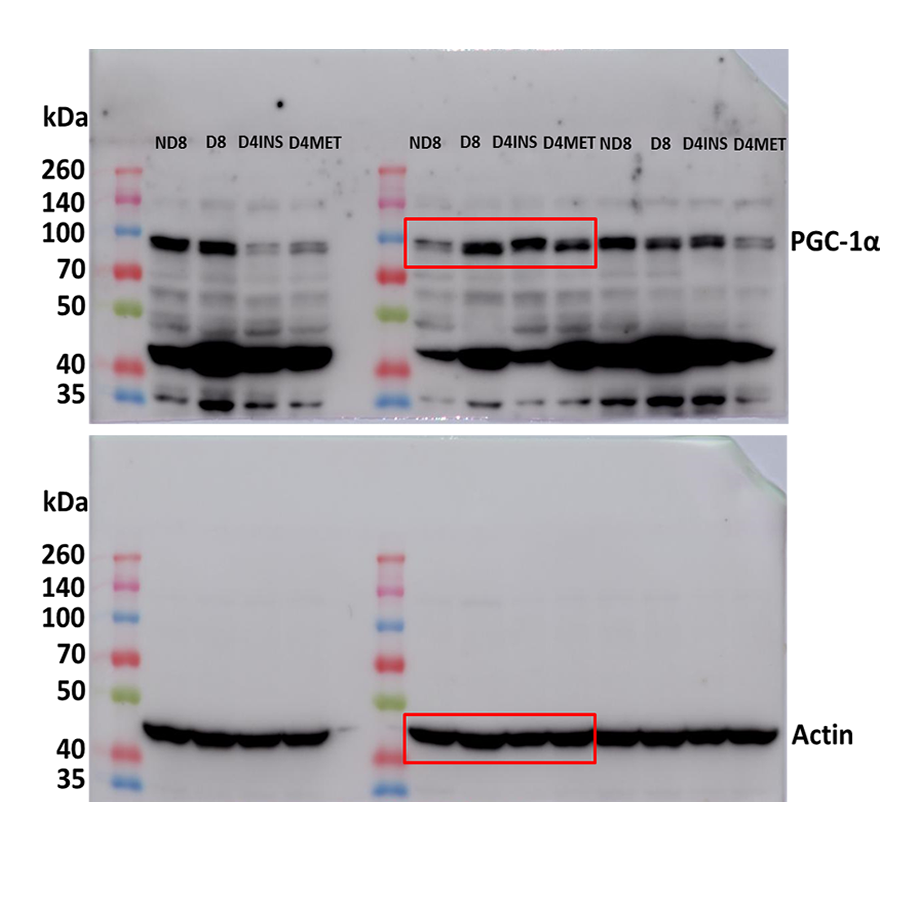


**Supplemental Figure 4.** Full-length blots of PGC-1α and actin expression for the long period experiment. The red boxes highlight the cropped bands shown in Figures 4B and 4C. PGC-1α was detected at 90 kDa and actin was detected at 42 kDa.


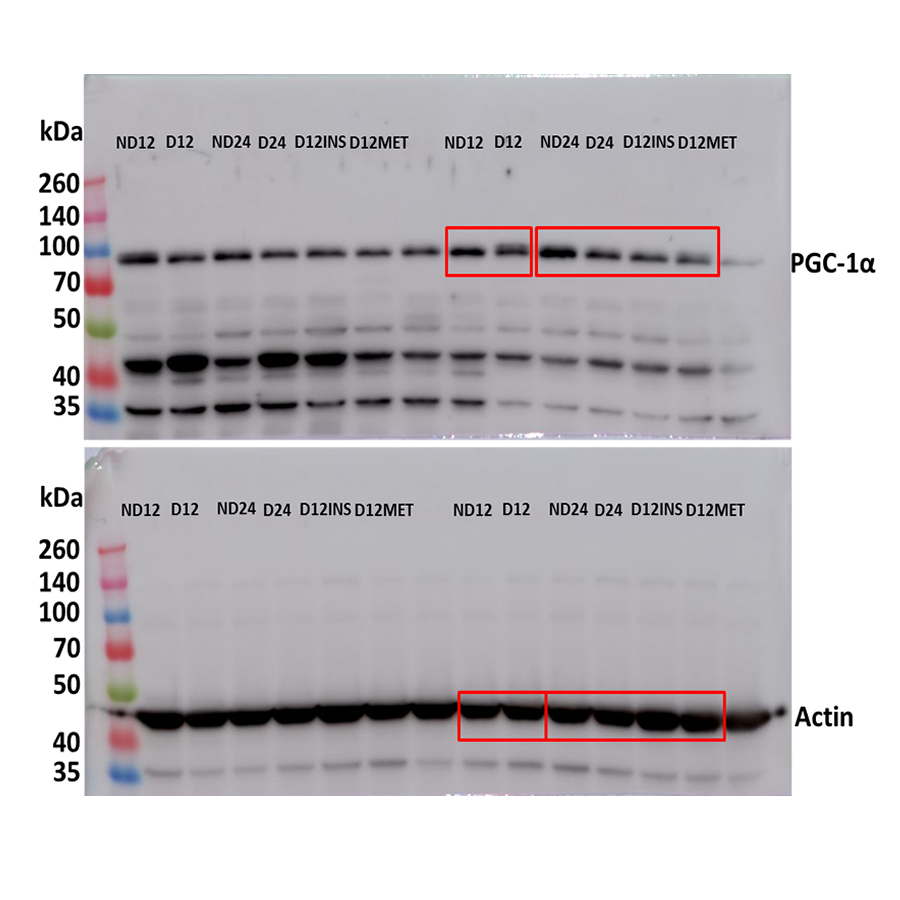

Supplement: Supplementary Information [file srep40544-s1.doc]
